# Day one postoperative MRI findings following electrode placement for deep brain stimulation: analysis of a large case series

**DOI:** 10.3389/fneur.2023.1253241

**Published:** 2023-12-19

**Authors:** Benjamin S. Succop, Carlos Zamora, Daniel Alberto Roque, Eldad Hadar, Brice Kessler, Carolyn Quinsey

**Affiliations:** ^1^School of Medicine, University of North Carolina at Chapel Hill, Chapel Hill, NC, United States; ^2^Department of Neuroradiology, University of North Carolina at Chapel Hill, Chapel Hill, NC, United States; ^3^Department of Neurology, University of North Carolina at Chapel Hill, Chapel Hill, NC, United States; ^4^Department of Neurosurgery, University of North Carolina at Chapel Hill, Chapel Hill, NC, United States

**Keywords:** deep brain stimulation, postoperative MRI, functional neurosurgery, stereotactic neurosurgery, Parkinson's disease, essential tremor

## Abstract

**Objective:**

This study sought to characterize postoperative day one MRI findings in deep brain stimulation (DBS) patients.

**Methods:**

DBS patients were identified by CPT and had their reviewed by a trained neuroradiologist and neurosurgeon blinded to MR sequence and patient information. The radiographic abnormalities of interest were track microhemorrhage, pneumocephalus, hematomas, and edema, and the occurrence of these findings in compare the detection of these complications between T1/T2 gradient-echo (GRE) and T1/T2 fluid-attenuated inversion recovery (FLAIR) magnetic resonance (MR) sequences was compared. The presence, size, and association of susceptibility artifact with other radiographic abnormalities was also described. Lastly, the association of multiple microelectrode cannula passes with each radiographic finding was evaluated. *Ad-hoc* investigation evaluated hemisphere-specific associations. Multiple logistic regression with Bonferroni correction (corrected *p* = 0.006) was used for all analysis.

**Results:**

Out of 198 DBS patients reviewed, 115 (58%) patients showed entry microhemorrhage; 77 (39%) track microhemorrhage; 44 (22%) edema; 69 (35%) pneumocephalus; and 12 (6%) intracranial hematoma. T2 GRE was better for detecting microhemorrhage (OR = 14.82, *p* < 0.0001 for entry site and OR = 4.03, *p* < 0.0001 for track) and pneumocephalus (OR = 11.86, *p* < 0.0001), while T2 FLAIR was better at detecting edema (OR = 123.6, *p* < 0.0001). The relatively common findings of microhemorrhage and edema were best visualized by T2 GRE and T2 FLAIR sequences, respectively. More passes intraoperatively was associated with detection of ipsilateral track microhemorrhage (OR = 7.151, *p* < 0.0001 left; OR = 8.953, *p* < 0.0001 right). Susceptibility artifact surrounding electrodes possibly interfered with further detection of ipsilateral edema (OR = 4.323, *p* = 0.0025 left hemisphere only).

**Discussion:**

Day one postoperative magnetic resonance imaging (MRI) for DBS patients can be used to detect numerous radiographic abnormalities not identifiable on a computed tomographic (CT) scan. For this cohort, multiple stimulating cannula passes intraoperatively was associated with increased microhemorrhage along the electrode track. Further studies should be performed to evaluate the clinical relevance of these observations.

## Introduction

Deep brain stimulation (DBS) involves the placement of high-frequency, stimulating electrodes in brain regions of clinical interest to mitigate the symptoms of neurologic disorders (1). DBS has become a widespread, flexible, and accessible treatment thanks to the development of implantable pulse generators, less invasive procedures, and segmented electrodes, among other advances (2, 3). DBS is now considered a frontline treatment for motor fluctuations and tremor in Parkinson's Disease (PD) and has also found extensive use in essential tremors, dystonia, and mood disorders refractory to pharmacological interventions (2, 4). Surgical complications can include symptomatic hemorrhage (~2% of cases), asymptomatic microhemorrhage, pneumocephalus, edema, seizure, infection, and malformation of electrode leads (<5% prevalence each) (5).

For many years, post-placement computed tomography (CT) has been used to confirm the placement of DBS leads and evaluate for the presence of surgical complications (6). While magnetic resonance imaging (MRI) is a superior method for anatomically localizing DBS leads (7, 8) and evaluating for the presence of microhemorrhages (9), it has been historically avoided due to concerns of radiofrequency-induced implant heating and tissue damage (10). Such concerns were based on simulation models as well as three verified adverse clinical events: two cases of DBS pulse generator failure and one case of temporary edema surrounding an electrode felt to develop during the acquisition of 1.5 T MRIs (11). Efforts to circumvent this challenge included a fusion of preoperative MRI with postoperative CT, allowing for improved visualization over postoperative CT alone (12). However, this fusion method still has shortcomings compared to obtaining true postoperative MRI, namely the greater predisposition to obstructive artifact, translational errors of up to 3 mm in positioning introduced during the fusion process, and insufficient sensitivity for identification of perielectrode soft tissue changes (13). Over time, a number of studies have demonstrated that postoperative MRI can be safely performed in DBS patients at 1.5 T and 3.0 T if specific, restricted conditions are met during scanning (14–18). These promising safety data have allowed for the incorporation and study of postoperative MRI in the care of DBS patients (19–22).

The existing postoperative DBS MRI literature has largely focused on evaluation of procedure safety (19–22), electrode mapping and clinical correlates (7, 23, 24), functional connectivity (25), electrode artifact (26, 27), and imaging findings such as symptomatic hemorrhage and edema (28–33). To date, there has been no comprehensive evaluation of postoperative radiographic abnormalities using MRI nor comparison of sequences best suited for detecting these phenomena. Furthermore, while symptomatic hemorrhage, edema, and artifact have been separately noted, no study has yet attempted to identify perioperative variables that contribute to these findings (27–33). Thus, the present study seeks to describe the rates of radiographic abnormalities, specifically intracranial hematoma, entry and track microhemorrhage, pneumocephalus, and edema, in day one postoperative patients using MRI, compare the detection of these radiographic abnormalities between T2 gradient-echo (GRE) and T2 fluid-attenuated inversion recovery (FLAIR) MR sequences, and evaluate the association of these complications with multiple intraoperative stimulating microelectrode passes and the presence of susceptibility artifact.

## Methods

### Participants and inclusion criteria

This institutional review board (IRB)-approved study (IRB 19-0726, Biomedical Institutional Research Board, Office of Human Research Ethics, UNC Chapel Hill) consisted of a retrospective analysis of DBS imaging and outcomes at a single institution. Possible subjects were identified by surgical CPT code (61868) (34) and consisted of adults who underwent bilateral DBS electrode implantation for any etiology at a single academic health care institution.

### DBS surgical workflow

At our institution, standard DBS surgical protocol involves two distinct surgical procedures. In the first procedure, intracranial leads are implanted bilaterally. This portion is typically performed in the patient's awake state accompanied by microelectrode recording and microelectrode stimulation to confirm therapeutic placement using a NeuroNav MER system. First, a single surgical track is made on one side according to preoperative surgical planning to reach the site of optimal stimulation. Prior to insertion of the lead, a cannula with a stimulating microelectrode is placed in the surgical track and advanced with awake microstomulation of the patient using the ring of the cannula to clinically identify the target that optimizes therapeutic relief with minimal side effects. If an optimal stimulation site is not found, the cannula with the stimulating microelectrode is repositioned within the track medially, laterally, anteriorly, or posteriorly and the process is repeated until optimal therapeutic effect is achieved. The stimulating microelectrode is then removed and the DBS lead is placed along the track into this position. This process is then repeated for the contralateral side. Placement is verified by intraoperative CT and desired clinical stimulation response. Postoperative MRI is collected on the first day after surgery. A secondary surgery is performed 1–2 weeks later consisting of placement of an implanted programmable generator with connection to the cranial leads.

### Perioperative variables

Chart review identified several perioperative variables of interest, including presence of multiple electrode passes, model of electrode, anatomical target, MRI sequences included in the obtained imaging, and clinical hemorrhages. Multiple passes are defined by medial/lateral and/or anterior/posterior repositioning of the stimulating microelectrode cannula with repeat advancement along the track when optimal therapeutic effect is not attained during awake stimulation in the original pass; this information was obtained from the operation note.

### MRI protocols

The detection of radiographic abnormalities was compared using two protocols in this study, both performed on a 1.5 T MRI scanner (Siemens, Erlangen, Germany). The first and current MRI protocol consists of a sagittal non-isovolumetric 3D T1 MP-RAGE (TR, 2200; TE, lowest; TI, 900; average, 1; flip angle, 8; field of view, 256; slice thickness, 1 mm; gap, 0 mm; time to acquisition) and axial T2 FLAIR (TR > 6000; TE, 330; average, 1.4; flip angle, variable; field of view, 230; slice thickness, 1 mm; gap, 0 mm). The second, previously used MRI protocol consisted of axial T1 GRE (TR, 50; TE, 7.36; average, 1; flip angle, 30; field of view, 240; slice thickness, 1 mm; gap, 0 mm) and coronal T2 GRE (TR, 700; TE, 15; average, 1; flip angle, 15; field of view, 240; slice thickness, 1 mm; gap, 0 mm).

### Image analysis

A team consisting of a neurosurgeon (CQ) and a neuroradiologist (CZ) blinded to the clinical variables, patient, and MR sequence reviewed and characterized the randomized MR scans across patients by consensus for the presence of radiographic abnormalities, including hemorrhage at the entry site or along the electrode track, edema, perielectrode susceptibility artifact, pneumocephalus, and intracranial hematoma. Hyperintensity with blooming <1 cm in diameter along the entry or track was characterized as entry or track microhemorrhage, respectively; hyperintensity without blooming around the electrode was characterized as edema; hyperintensity with blooming >1 cm in diameter was characterized as hematoma, which combined subarachnoid hemorrhages, subdural hematomas, intraventricular hemorrhages, and intraparenchymal hemorrhages; and extra-axial hypointensity was characterized as pneumocephalus. In the case of disagreement, a neurologist (DR) on the team separately was presented the images in an identically blinded manner, with the majority determination being reported. Artifact was identified as distortion around the electrode; asymmetric artifact referred to artifact that was >1 cm in diameter than its contralateral counterpart. The presence or absence of these variables was assigned the binary values of one and zero, respectively, for the purpose of multiple logistic regression.

### Statistics

All statistical calculations and analyses were conducted in Graph Prism 10. Summary statistics were generated for the imaging outcomes and perioperative chart variables based on their frequency. Logistic regression was used to identify significant associations between T2 GRE, T2 FLAIR, presence of multiple passes, and each of the imaging complications described above. *Ad-hoc*, multiple logistic regressions were used to evaluate the effect of hemisphere-specific multiple passes compared to a single pass and asymmetrically larger susceptibility artifact on ipsilateral entry microhemorrhage, track microhemorrhage, and edema. In this investigation, a total of eight logistic regressions were evaluated; therefore, to counteract the problem of multiple comparison, a Bonferroni correction was applied to the typical significance threshold of *p* = 0.05 for all comparisons to yield a corrected significance threshold of *p* = 0.006. No random effects were considered.

## Results

### Summary data

In total, 198 patients were analyzed. Of those, 180 patients (91%) were undergoing treatment for Parkinson's, 10 patients (5%) were undergoing treatment for essential tremor and eight (4%) were undergoing treatment for other conditions. While 176 patients (89%) underwent targeting of the subthalamic nucleus, in 12 patients (6%) the ventralis intermedius nucleus was targeted, in 10 patients (5%) the globus pallidus internalis was targeted, and in four patients (2%) the ventral palladium was targeted (2%). With respect to imaging protocols, 57 patients (29%) were put through the T2 FLAIR protocol and 141 patients (71%) were subjected to the T2 GRE protocol (Table 1).

**Table 1:** Summary data^*^.

**Variable**	**Patients N (%)**	**Left hemisphere N (%)**	**Right hemisphere N (%)**
Sample size	198 (100%)	198 (100%)	198 (100%)
Medtronic 3389 electrode	195 (98%)	195 (98%)	195 (98%)
Medtronic 3391 electrode	3 (2%)	3 (2%)	3 (2%)
STN target	176 (89%)	176 (89%)	176 (89%)
VIM target	11 (6%)	11 (6%)	11 (6%)
GPI target	9 (5%)	9 (5%)	9 (5%)
Ventral palladium target	3 (2%)	3 (2%)	3 (2%)
Parkinson	180 (91%)	—	—
Essential tremor	9 (5%)	—	—
Other pathology	8 (4%)	—	—
Revision	31 (16%)	20 (10%)	15 (6%)
Multiple passes	60 (30%)	32 (16%)	32 (16%)
T2 GRE	141 (71%)	—	—
T2 FLAIR	57 (29%)	—	—
Asymmetrically larger susceptibility artifact (>1 cm)	165 (83%)	89 (45%)	76 (38%)
Entry hemorrhage	115 (58%)	73 (37%)	71 (36%)
Edema	43 (22%)	22 (11%)	27 (14%)
Pneumocephalus	69 (35%)	—	—
Track hemorrhage	78 (39%)	47 (23%)	50 (25%)
Hematoma	10 (5%)	4 (2%)	6 (3%)
Subdural hematoma	4 (2%)	2 (1%)	2 (1%)
Subarachnoid hemorrhage	6 (3%)	4 (2%)	2 (1%)
Intraventricular hemorrhage	1 (1%)	1 (1%)	0 (0%)

* All patients underwent bilateral DBS. Where relevant, hemisphere-specific data is reported.

Overall, 164 patients (83%) manifested asymmetric electrode susceptibility artifact, 115 patients (58%) demonstrated entry microhemorrhage, 77 patients (39%) exhibited track microhemorrhage, 44 patients (22%) had edema in tissue adjacent to the electrode, 69 patients (35%) exhibited pneumocephalus, and 12 patients (6%) showed an intracranial hematoma on imaging (Table 1).

### Outcome analysis

Multiple logistic regression was performed for evaluating the association of radiographic abnormalities with each MR sequence, multiple passes with the stimulating microelectrode cannula, and susceptiblity artifact, demonstrated in Table 2 with the odds ratio and *p*-values listed underneath proportions for each. Entry hemorrhage was significantly associated with T2 GRE (OR = 14.82, *p* < 0.0001) and presence of multiple passes (OR = 4.03, *p* < 0.0001). Track microhemorrhage was significantly better visualized with T2 GRE (OR = 7.61, *p* < 0.0001) and presence of multiple passes compared to a single pass (OR = 7.91, *p* < 0.0001) (Table 2). Pneumocephalus was significantly associated with T2 GRE (OR = 11.86, *p* < 0.0001). Edema was significantly associated with T2 FLAIR (OR = 123.6, *p* < 0.0001). Intracranial hematoma was also significantly associated with T2 FLAIR (OR = 8.625, *p* = 0.0004). The presence of artifacts had no significant associations with any perioperative variable (Table 2).

**Table 2:** Comparing radiographic abnormalities by MR sequence and number of passes.

**Group**	**Sample size**	**Susceptibility artifact**	**Entry microhemorrhage**	**Track microhemorrhage**	**Edema**	**Pneumocephalus**	**Macrohemorrhage**
Patient (*N*)	198	165	115	78	43	69	10
(%)		83%	58%	39%	22%	35%	8%
T2 GRE (n)	140	111	105	71	3	65	4
(%)		79%	75%	51%	2%	46%	3%
Odds ratio		0.28	14.82	7.61	0.01	11.86	0.13
(*p*-value)		0.0104	< 0.0001	< 0.0001	< 0.0001	< 0.0001	0.0002
T2 flair (n)	55	51	10	6	40	3	11
(%)		93%	18%	11%	73%	5%	20%
Odds ratio		3.27	0.08	0.12	123.6	0.07	8.63
(*p*-value)		0.0341	< 0.0001	< 0.0001	< 0.0001	< 0.0001	0.0004
Single pass (n)	138	114	68	35	34	47	6
(%)		82%	49%	25%	25%	34%	4%
Odds ratio		0.71	0.25	0.13	1.82	0.87	1.19
(*p*-value)		0.4252	< 0.0001	< 0.0001	0.1341	0.6642	0.7705
Multiple passes (n)	60	51	47	43	9	22	4
(%)		85%	80%	73%	15%	37%	7%
Odds ratio		1.41	4.03	7.91	0.55	1.15	0.84
(*p*-value)		0.4252	< 0.0001	< 0.0001	0.1341	0.6642	0.7705

### *Ad-hoc* hemisphere-specific outcome analysis

*Ad-hoc* analysis sought to present a hemisphere-specific investigation of associations, in which the inputs were hemisphere-specific single or multiple passes and hemisphere-specific asymmetrically larger artifact (Table 3). The outputs were hemisphere-specific entry microhemorrhage, track microhemorrhage, and edema. Neither left entry nor right entry microhemorrhage was significantly associated with multiple passes or asymmetrically larger artifact. Microhemorrhage was associated with multiple passes on the same hemisphere (Left OR = 7.151, *p* < 0.0001; Right OR = 8.953, *p* < 0.0001). In patients with detected unilateral edema, there was an inverse association with presence of asymmetric artifact on the same hemisphere (OR = 4.323, *p* = 0.0025).

**Table 3:** Comparing findings by hemisphere (*ad-hoc*).

**Group**	**Sample size**	**Left entry microhemorrhage**	**Right entry microhemorrhage**	**Left track microhemorrhage**	**Right track microhemorrhage**	**Left edema**	**Right edema**
Patients (*N*)	198	73	71	47	50	22	27
(%)		37%	36%	24%	25%	11%	14%
Multiple passes left	32	14	16	19	8	6	4
(%)		44%	50%	59%	25%	19%	13%
Odds ratio		1.69	1.538	7.151	0.9762	2.149	0.8
(*p*-value)		0.1868	0.2675	< 0.0001	0.9568	0.1622	0.6933
Multiple passes right	32	13	20	7	21	2	1
(%)		41%	63%	22%	66%	6%	3%
Odds ratio		1.446	2.842	0.875	8.953	0.4833	0.1578
(*p*-value)		0.3526	0.0089	0.7738	< 0.0001	0.344	0.075
Artifact left	110	28	39	28	23	5	20
(%)		25%	35%	25%	21%	5%	18%
Odds ratio		0.4548	0.6102	1.255	0.6042	0.2008	2.386
(*p*-value)		0.0103	0.0911	0.5028	0.1262	0.0025	0.0497
Artifact right	73	27	31	20	21	15	3
(%)		37%	42%	27%	29%	21%	4%
Odds ratio		1.279	1.092	1.356	1.323	4.323	0.2296
(*p*-value)		0.4271	0.7678	0.372	0.4027	0.0025	0.0087

## Discussion

This study describes several radiographic abnormalities, namely entry microhemorrhage, track microhemorrhage, edema, pneumocephalus, and intracranial hematoma present on postoperative day one in DBS patients, compares the efficacy of T2 GRE and FLAIR in identifying them, and evaluates associations with the presence of multiple passes and susceptibility artifact. As expected, T2 GRE was more associated with the detection of entry hemorrhage, track hemorrhage, and air, while T2 FLAIR was more associated with the detection of edema. GRE is optimized for detecting microhemorrhages due to its greater sensitivity to magnetic susceptibility artifacts compared to other non-GRE MRI sequences (35). T2 FLAIR is, by definition, fluid attenuated on account of the manipulation of the inversion time to negate the contribution of cerebrospinal fluid (CSF) to the signal, thus allowing characterization of fluid collections and edema (36).

### Microhemorrhage

MRI has been demonstrated to be superior to CT in the detection of microhemorrhages (9), and our study demonstrates that these phenomena are a common occurrence in day one postoperative DBS patients. Of note was the novel observation by the *ad-hoc* analysis that multiple cannula passes during the procedure was associated with increased likelihood of microhemorrhage detected along the unilateral track but not at the unilateral entry site. This difference between entry and track hemorrhage is reasonably intuitive, as making another pass consists of repositioning the cannula within the burr hole, not opening a new one. Thus, one possible adverse effect of not achieving ideal therapeutic effects with preoperative targeting coordinates and readjusting mid-procedure is the consequent greater degrees of microhemorrhage along the track. The increased likelihood of this track microhemorrhage is likely due to capillary injury along the track caused by repositioning of the cannula. Awake DBS practitioners should consider the increased risk of track microhemorrhage from multiple passes when undergoing preoperative target planning and intraoperative localization. However, it remains unclear whether there is any clinical significance to greater degree of microhemorrhage, as our study did not consider long-term outcomes.

### Edema

One phenomenon that has been studied previously in the literature was the presence and characteristics of vasogenic edema. This is a unique DBS radiographic phenomenon that thus far appears to have no functional impact on patients, though more investigation is needed (28–33). Interestingly, the prevalence of vasogenic edema varies widely across the studies reviewed, ranging from 6.3% to 100% of imaging, with a mean prevalence calculated at 25.6%. In addition to sequence selection, the variability across the literature can likely be attributed to several factors. Firstly, the time from procedures to scan varied drastically, from immediate postoperative imaging to up to 6 months after, and the studies reviewed tended to show a decreased prevalence as time from procedure increased, suggesting that the edema is part of the typical course of postoperative healing (28–33). Secondly, thresholds for categorizing electrode trajectory T2 FLAIR hyperintensities as edema likely differed across studies.

Additionally, this study's *ad-hoc* hemisphere-specific analysis illustrated a significant association between the presence of susceptibility artifact on the one hand and the detection of edema on the other. One possible interpretation of this finding is that the electrode artifact obscures the detection of ipsilateral edema. Given that electrode-related susceptibility artifact was prevalent throughout the imaging reviewed in this study, there could have been a significant confounding effect in the detection of edema and hemorrhage around the electrodes. It has been reported in the literature that the size of such artifact is proportional to the increase in the angle between the long axis of the electrode and the direction of the main magnetic field (37). Steps should be taken in patient positioning and image processing to allow for a better detection of the day one post-surgical changes discussed in this study that are identifiable on MRI.

### Other imaging complications

Subarachnoid, intraventricular, and subdural hemorrhages are rare in DBS, reported in 6.3% of patients from studies based on MRI, which is consistent with our study (28). The presence of pneumocephalus was relatively common, being found in nearly half of our patients, though the true proportion may have been higher since some of our sequences did not routinely include the vertex, a frequent site of pneumocephalus. However, existing literature has demonstrated that, while implicated in brain shift, intracranial air does not impact electrode position and negligibly impacts patient outcomes (38, 39).

### Limitations

The principal limitations of this study were its design as a retrospective analysis and the *ad-hoc* nature of the hemisphere-specific analysis. Furthermore, clinical outcomes were not evaluated longitudinally, so the clinical impacts of the MRI findings studied, particularly microhemorrhage and edema, could not be determined from our data. There is a possibility that images were miscategorized by the blinded analysts, reducing the accuracy of our results. Lastly, the presence of susceptibility artifact may have obscured the detection of the complications that were analyzed, particularly those that were near in proximity to the electrodes.

## Conclusion

This study represents an attempt to characterize DBS day one postoperative MRI abnormalities including microhemorrhage, edema, pneumocephalus, and intracranial hematoma as well as evaluate the association of these phenomena with select perrioperative variables. Several noteworthy findings were observed. Firstly, microhemorrhage along the track and entry site is a common occurrence, the detection of which is an advantage of postoperative MRI over CT. Secondly, multiple cannula passes were associated with increased likelihood of microhemorrhage detected along the unilateral track. Lastly, edema detection was inversely associated with ipsilateral susceptibility artifact, which raises the question of whether or not such artifact obscures the detection of edema and other complications identifiable on imaging. The early postoperative MRI findings of microhemorrhage and edema identified in this study are possibly immediate sequelae of the procedure, but further investigation is needed to definitively evaluate the longitudinal effects of these day one postoperative imaging findings. Additionally, further studies are necessary to characterize whether microhemorrhage is clinically significant and to minimize the presence of susceptibility artifact.

## Data availability statement

The raw data supporting the conclusions of this article will be made available by the authors, without undue reservation.

## Ethics statement

The studies involving human participants were reviewed and approved by the University of North Carolina (UNC) Office of Human Research Ethics Institutional Research Board (approval number 19-0726). Written informed consent for participation was not required for this study in accordance with the national legislation and the institutional requirements.

## Author contributions

BS completed IRB, led data analysis, conducted statistics, and drafted with manuscript. CZ evaluated postop imaging and characterized findings, assisted with manuscript, and MRI expertise. DR assisted with data analysis specifically deciding characterization of inconclusive imaging, drafting of manuscript, and neurological expertise. EH assisted with IRB, patient data request, drafting of manuscript, and neurosurgical expertise. BK assisted with drafting of manuscript and neurosurgical expertise. CQ assisted with IRB, data analysis, drafting of manuscript, and neurosurgical expertise. All authors contributed to the article and approved the submitted version.
